# The role of hormones on *Toxoplasma gondii* infection: a systematic review

**DOI:** 10.3389/fmicb.2014.00503

**Published:** 2014-10-09

**Authors:** María de la Luz Galván-Ramírez, Adrián Fernando Gutiérrez-Maldonado, Fabiola Verduzco-Grijalva, Judith Marcela Dueñas Jiménez

**Affiliations:** Neurophysiology Laboratory, Department of Physiology, Health Sciences University Center, University of GuadalajaraGuadalajara, Jalisco, Mexico

**Keywords:** *Toxoplasma* infection, steroids hormones, no steroid hormones, toxoplasmosis, *Toxoplasma*

## Abstract

**Background:** *Toxoplasma gondii* is the causal agent of toxoplasmosis in which one third of the world's population has been infected. In pregnant women, it may cause abortion and severe damage to the fetal central nervous system. During pregnancy, the prevalence of toxoplasmosis increases throughout the second and third quarter of gestation, simultaneously progesterone and 17β-estradiol also increase. Thus, it has been suggested that these hormones can aggravate or reduce parasite reproduction. The aim of this study was reviewing the relationship between hormones and infection caused by *T. gondii* in several experimental animal models and humans, focused mainly on: (a) congenital transmission, (b) parasite reproduction, (c) strain virulence, (d) levels of hormone in host induced by *T. gondii* infection, and (e) participation of hormone receptors in *T. gondii* infection. Are the hormones specific modulators of *T. gondii* infection? A systematic review methodology was used to consult several databases (Pub Med, Lilacs, Medline, Science direct, Scielo, Ebsco, Sprinker, Wiley, and Google Scholar) dated from September, 2013 to March, 2014.

**Results:** Thirty studies were included; eight studies in humans and 22 in animals and cell cultures. In the human studies, the most studied hormones were testosterone, progesterone, prolactin, and 17β-estradiol. Type I (RH and BK) and Type II (Prugniaud, SC, ME49, T45, P78, and T38) were the most frequent experimental strains.

**Conclusions:** Thirty-five years have passed since the first studies regarding *T. gondii* infection and its relationship with hormones. This systematic review suggests that hormones modulate *T. gondii* infection in different animal models. However, given that data were not comparable, further studies are required to determine the mechanism of hormone action in the *T. gondii* infectious process.

## Introduction

*Toxoplasma gondii* (*T. gondii*) is the causal agent of toxoplasmosis and one third of the world population has been affected by this parasite (el-On and Peiser, 2003). In immunocompetent adults, 80% of the cases can be asymptomatic. On the other hand, in immunocompromised patients, *T. gondii* is an opportunistic parasite that has been held responsible for mortal encephalitis (Cabrera-Muñoz et al., 2010).

Congenital transmission of *T. gondii* causes severe consequences in which the degree of damage depends on the time when the mother is infected (Speroff et al., 1999). Infection during early pregnancy can result in apoptosis of placental cells and fetal resorption (Senegas et al., 2009). When pregnant females infected during latter stage of pregnancy and inflammatory responses are low, congenital transmission is likely to occur (Roberts et al., 2001; Pfaff et al., 2008). The transmission frequency of *T. gondii* is high (80%) at end of pregnancy.

### Pregnancy and *T. gondii* infection

During pregnancy, maternal hormones alter the immune responses of the mother in the presence of fetal antigens. The increases in the susceptibility to infection and a diminished pro-inflammatory response have critical anti-parasitic properties that cause an unfavorable development of toxoplasmosis (Craig et al., 2001; Roberts et al., 2001; Prigione et al., 2006; Dionne et al., 2012). In the second and third trimester of gestation, there is a significant increase of 17β-estradiol and progesterone levels and it is during this period, when the prevalence of *Toxoplasma* infection increases (Montoya and Remington, 2008; Al-warid and Al-qadhi, 2012).

### 17β-estradiol and *T. gondii* infection

17β-estradiol (E2) is synthetized mainly in the ovary, breast, endometrial tissue, and brain. E2 plays a vital role in the menstrual cycle and human reproduction. In the nervous system, the estrogens are neuroprotective (Duenas et al., 1996; Arevalo et al., 2010). It has been reported that the administration of pharmacological doses of 17β-estradiol increases the susceptibility to *Toxoplasma* infection (Pung and Luster, 1986).

### Progesterone

Progesterone is present in the ovary and corpus luteum where it is primarily involved in the second phase of the menstrual cycle and reproductive processes of women. Progesterone is synthetized in breast, endometrial, and brain too (Speroff et al., 1999). In cells infected with tachyzoites of *T. gondii*, progesterone did not regulate the replication of parasites (Gay-Andrieu et al., 2002). Progesterone levels are reduced during pregnancy in sheep after infection by *T. gondii* (Aiumalamai et al., 1990; Fredriksson et al., 1990).

### Testosterone levels regulation by *T. gondii* infection in human beings and mice

Testosterone and their derivatives (dihydrotestosterone and dehydroepiandrosterone) are androgens produced mainly in male gonads, adrenal glands and the brain. Testosterone can act directly as a ligand of androgen receptors (AR) found in several target tissues. Androgens stimulate the development of the secondary sexual characters in males, participate in human reproduction and maturation of human fetal testes (O'Shaughnessy and Fowler, 2014). In the brain, it is considered as a neuroprotective hormone (Kurth et al., 2014). IgG anti-*Toxoplasma* antibodies were significantly correlated to testosterone (Shirbazou et al., 2011), and results are different accord type strain (Kaňková et al., 2011). *T. gondii* produces high testosterone levels in infected animals and mRNA expression of luteinizing hormone receptor (LHR) (Oktenli et al., 2004; Abdoli et al., 2012; Lim et al., 2013).

### Thyroxine (T4) and *T. gondii* infection

Studies in Nylar female mice infected with *T. gondii*, exhibited hypogonadotrophic hypogonadism secondary to hypothalamic dysfunction (Stahl et al., 1985, 1994). These mice infected with *T. gondii* Cornell strain, present atrophy in the thymus, ovaries, and uterus, cessation of cycling, anovulation, and decline of serum thyroxine (T4) levels (Stahl et al., 1985).

### Corticosteroids effect on *T. gondii*

Cortisol is a glucocorticoid hormone secreted by the adrenal cortex. It works through a signal transduction pathway that initiates by hormone linkage to specific cell receptors. Proteins synthesized by the glucocorticoid response inhibit or stimulate the specific tissue (Gardner et al., 2011). Cortisone increased the amount of tachyzoites, cysts and cystozoite, as the breakage of cysts released a higher resistant antigen-cystozoite in mice brains infected with *T. gondii* (Hulínská et al., 1990).

### Anti-parasitic effect of prolactin on *T. gondii* infection

PRL is capable of inhibiting multiplication of *Toxoplasma* in murine microglial cell cultures (Benedetto et al., 2001). PRL significantly restricted intracellular growth of *Toxoplasma* in mice and human cell lines (Dzitko et al., 2010; 2012). Moreover, it been documented that women with hyperprolactinemia showed lower *T. gondii* prevalence (Dzitko et al., 2008). It has been reported that serum human prolactin (shPRL) has the capacity to bind to live RH tachyzoites (type I) and ME49 (type II) strains in a specific way (Dzitko et al., 2013).

The aim of this study was to review the relationship between hormones and infection by *T. gondii* in several experimental animal models and humans. Focusing the information on: (a) congenital transmission, (b) parasite reproduction, (c) strain virulence, (d) levels of hormone in host induced by *T. gondii* infection, (e) participation of hormone receptors in *T. gondii* infection.

## Materials and methods

### Database search

Reports from September 2013 to February 2014 were obtained from a total of nine databases (Pub Med, Lilacs, Medline, Science direct, Scielo, Ebsco, Sprinker, Wiley, Google Scholar). Mesh terms were “*Toxoplasma* or toxoplasmosis or *Toxoplasma gondii*” combined with progesterone, 17β-estradiol, testosterone, cortisol, cortisone, aldosterone, 11-desoxicorticosterone, dihydrotestosterone, dehydroepiandrosterone, and non-steroid hormones; growth hormone, prolactin, parathyroid hormone, corticotrophin, insulin, glucagon, luteinizing hormone, thyroid stimulating hormone, human chorionic gonadotropin, antidiuretic hormone, oxytocin, melanocyte stimulating hormone, somatostatin, thyrotropin-releasing hormone, gonadotropin-releasing hormone, noradrenaline, adrenaline, melatonin, thyroxine, and triiodothyronine. *Toxoplasma* and hormones and strain *Toxoplasma*. The criteria used for including data were: the full text of papers written in English (reviews and case reports not considered), studies performed on humans, animals, and in cell cultures.

### Data collection methods

**Figure 1:**
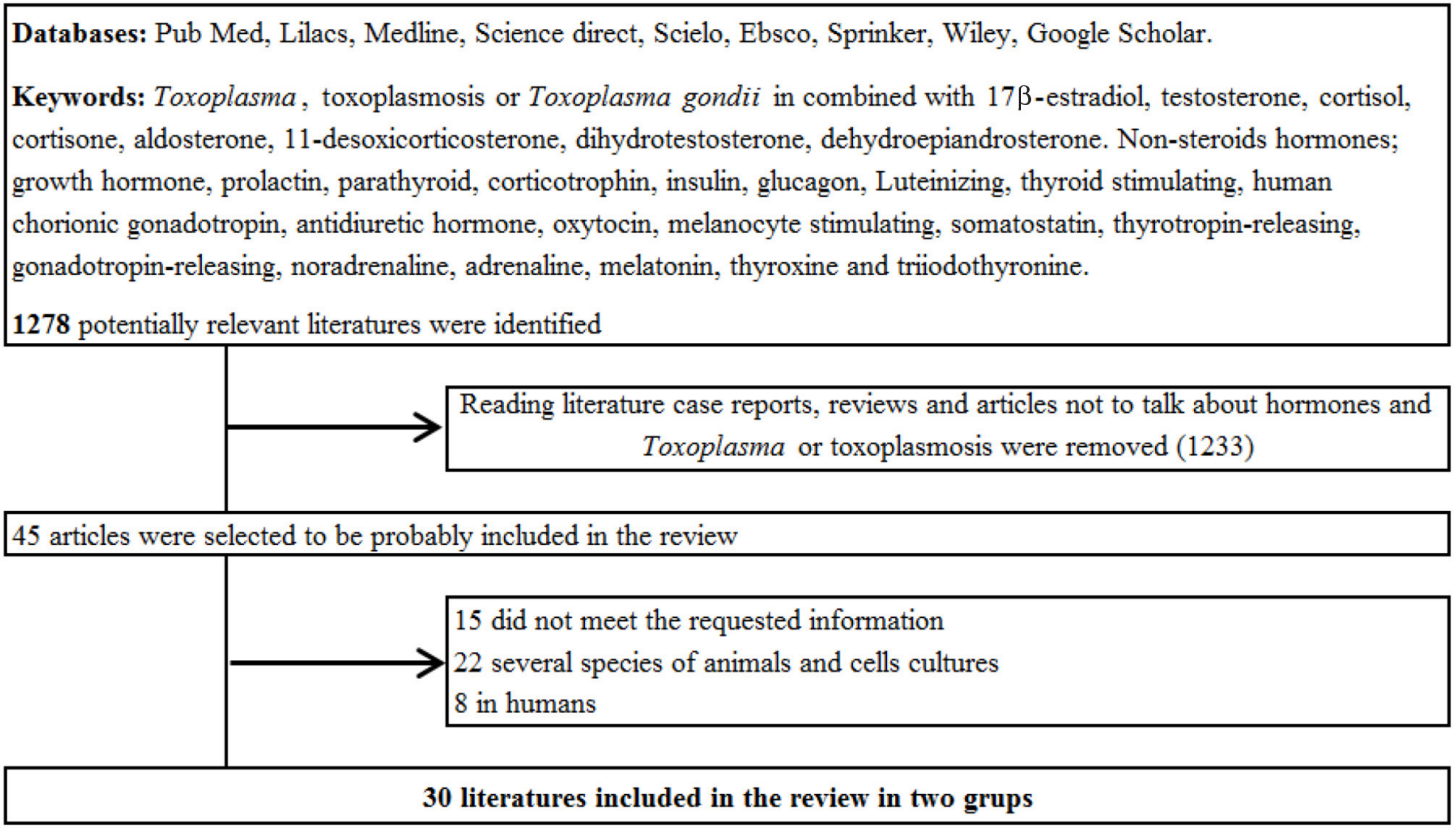
**This figure shows the flow of the search obtained, data collection methods, and database search**.

Two reviewers (GRML and GMAF) carefully studied all selected studies. The full text of selected original articles were obtained and reviewed. Inclusion criteria for this analysis were explicit data of all independent variables and at least one dependent variable; data collection and criteria eligibility were established for determining the frequency or proportion of each study. The independent variables were *T. gondii* strain, hormones, study design, stage of infection and developmental stage of the parasite, post infection evaluation time, age, host, and technical analysis. Dependent variables were increased or decreased of infection and number of parasites. Reference lists of full-text publications were examined for identifying studies not originally selected Figure 1.

From 30 articles meeting inclusion criteria, all results were captured on an Excel database. A number of studies presented frequency distribution of dependent variables; in these cases, the sum of the products of each value by frequency was included for comparison in the database. Some articles presented ranges, mean plus standard deviation; these articles were included in the database using the median.

## Results

**Figure 2:**
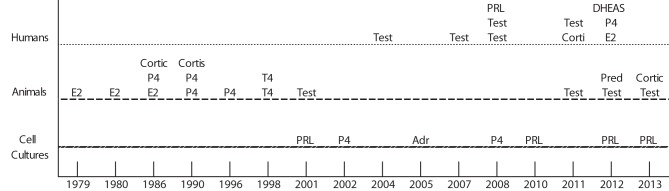
**Show studies development in human, animals and cells cultures with different hormones during a time period since 1979–2013.** E2, 17β-estradiol; Cortic, Corticosterone; Cortis, Cortisone; Corti, Cortisol; P4, Progesterone; T4, Thyroxine; Test, Testosterone; PRL, Prolactin; Adr, Adrenaline; DHEAS, Dehydroepiandrosterone; Pred, Prednisolone.

One thousand two hundred and seventy eight articles potentially related to *T. gondii* or hormones were found. However, only 45 were selected and of these, 30 met the inclusion criteria for this systematic review. The analysis was divided into three categories: (A) humans in Table 1, (B) several species of animals in Table 2, and (C) Cell cultures in Table 3 and studies conducted in the time period that this research included Figure 2.

**Table 1:** **Effect of hormones on *Toxoplasma gondii* infection in humans**.

**References**		**Age of the host (years)**	**Sex^a^**	**Analysis technique^b^**	**Hormones^c^**	**Diagnostic/group^d^**	***N***	**Results^e^**			***p***
1	Oktenli et al., 2004	17–18	NS	ELISA	Testosterone		*T. gondii* antibodies				
						Control	20	IgM:0.53 ± 0.13	IgG:0.43 ± 1.08	–	
						Normal testosterone levels	31	IgM:3.88 ± 1.14^*^	IgG:4.95 ± 0.91^*^	**↑**	**<0.001**
						Low testosterone levels	9	IgM:4.00 ± 1.03^*^	IgG:4.50 ± 1.08^*^	**↑**	**<0.001**
				QUIL			Total Testosterone (TT) nM/L ± SD				
						Control	20	17.11 ± 1.01		–	
						Normal testosterone levels	31	17.29 ± 1.38		–	
						Low testosterone levels	9	4.57 ± 0.56^*^		↓	**<0.001**
2	Hodková et al., 2007	21–24	NS	ELISA	Testosterone		*T. gondii* antibodies				
							89	Positive: 18		–	
								Negative: 71		–	
				Dom S			Dominance score				
						Infected	18	0.184^*^		↑	=0.051
						Uninfected	71	−0.57^*^			
							Masculinity score				
				Mas S		Infected	18	0.17		↑	0.17
						Uninfected	71	−0.03			
3	Flegr et al., 2008a			RIA	Testosterone		Testosterone levels ng/mL				
		21.03	W				174	0.230^*^		**–**	**<0.0001**
		20.91	M				91	0.387^*^		**–**	
				ELISA			*T. gondii* antibodies				
			W				174	Positive: 29		–	
			M				91	**Negative: 23**		**↑**	**<0.001**
4	Dzitko et al., 2008			ELISA	Prolactin		Seropositive *anti-Toxoplasma* antibodies				
		NS	W			Control	205	93			
			M			Control	76	39			
			W			Hyperprolactinaemia	168	57^*^		**↓**	**=0.025**
			M			Hyperprolactinaemia	66	31			
			W			Hypoprolactinaemia	32	10			
			M			Hypoprolactinaemia	9	3			
5	Flegr et al., 2008b			RIA	Testosterone		Testosterone levels nM/L				
		21.05	W				135	**0.23**		**–**	**<0.001**
		20.94	M				106	0.41		–	
							Digit radio 2D:4D				
			W	ELISA			194	Right: 0.315	Left: 0.587		
			M				106	**Right: 0.167**	Left: 0.002^*^	**–**	**<0.01**
6	Shirbazou et al., 2011			ELISA			Seropositive *T. gondii* antibodies				
		NS	W				73	24		–	
		NS	M				107	39		–	
							Cortisol levels in blood				
		NS	W		Cortisol	Uninfected	12			–	
		NS	M			Uninfected	19			–	
		NS	W			Infected	24	t: 5.774^*^			**<0.0001**
		NS	M			Infected	39			–	
							Testosterone levels in blood				
		NS	W		Testosterone	Uninfected	12			–	
		NS	M			Uninfected	19			–	
		NS	W			Infected	24	t: 2.491^*^			**=0.002**
		NS	M			Infected	39			–	
7	Al-warid and Al-qadhi, 2012			ELISA			Anti-*Toxoplasma* antibodies				
		19–40	W			Uninfected	9	(−) IgG	(−) IgM	–	
						Acute	10	(−) IgG	(+) IgM	–	
						Sub-acute	9	(+) IgG	(−) IgM	–	
						Chronic	13	(+) IgG	(+) IgM	–	
							Progesterone levels ng/dL ± SD				
			W	ELISA	Progesterone (P4)	Uninfected	9	18.3 ± 9.84			
						Infected	32	11.19 ± 9.76			
							P4 levels ng/dL ± SD				
						Acute	10	5.35 ± 7.15		–	
						Sub-acute	9	15 ± 9.01		–	
						Chronic	13	14.62 ± 10.38		–	
							Estradiol levels pg/dL ± SD				
			W	ELISA	17β-estradiol (E2)	Uninfected	9	53.61 ± 76.24		–	
						Infectadas	32	88.19 ± 101.10		–	
							E2 levels pg/dL ± SD				
						Acute	10	70.66 ± 51.08		–	
						Sub-acute	9	92.51 ± 78.70		–	
						Chronic	13	108.02 ± 138.67		–	
8	de la Torre et al., 2012	20–29		ELISA	DHEAS		Seropositive *T. gondii* antibodies				
							82	42			
				IL			DHEAS levels ug/dL				
			W			Active RC by *T. gondii*	26	58		–	
			M					206			
			W			RS of RC by *T. gondii*	19	95^*^			=0.12
			M					199^*^			=0.79
			W			Positive of *T. gondii w* OL	16	113			
			M					177			
			W			Negative assay for *T. gondii*	21	122^*^			=0.3
			M					161^*^			=0.87

a *M, Men; W, Woman; NS, Not Specified*.

b *ELISA, Enzyme-Linked ImmunoSorbent Assay; (QUIL), Chemiluminescence; Dom S, Dominance Score; Mas S, Masculinity Score; RIA, Radioimmunoassay; IL, Immunoluminimetric*.

c DHEAS, Dehydroepiandrosterone Sulphated; E2, 17β-estradiol; P4, Progesterone.

d *RS, Retinal Scars; RC, Retinochoroiditis; w OL, Without Ocular Lesions*.

e **↑**, Increased infection; **↓**, Decrement infection; ↑, Increased hormone; ↓, Decrement hormone;

* and bold, Statistically Significant. NS, Not specified.

**Table 2:** **Effect of *Toxoplasma gondii* infection on hormones in animals**.

**References**		**Type of study**	**Type of host**	**Age of the host (weeks)**	**Way of infection^a^**	**Stage parasite**	**Strain^b^**	**Number of parasites**	**Days post-infection**	**Analysis technique^c^**	**Hormones^d^**	**Group^e^**	***N***	**Results^f^**		***p***
1	Kittas and Henry, 1979	*In vivo*	Guinea-pigs	NS	SC	Cysts	Bk	50	42	Number of *To****i****xoplasma* cysts ± SD						
										HIS	17β-estradiol (E2)	Control F:	8	88.75 ± 21.60		
												Control M:	8	82.50 ± 21.1^*^		**<0.001**
												Gdt F:	8	63.00 ± 16.5	**↓**	
												Gdt M:	8	65.25 ± 10.8	**↓**	
												Gdt + Hex F:	8	200.25 ± 16.00	**↑**	
												Gdt + Hex M:	8	184.00 ± 36.80	**↑**	
2	Kittas and Henry, 1980	*In vivo*	Mice	11	SC	Cysts	Bk	30	42	Number of *Toxoplasma* cysts ± SD						
										HIS	17β-estradiol	Control F:	8	222 ± 42		
											(E2)	Control M:	8	220 ± 23		
												Gdt F:	8	189 ± 22^*^	**↓**	**<0.001**
												Gdt M:	8	178 ± 24^*^	**↓**	**<0.001**
												Gdt + Hex F:	8	598 ± 64^*^	**↑**	**<0.001**
												Gdt + Hex M:	8	599 ± 45^*^	**↑**	**<0.001**
3	Pung and Luster, 1986	*In vivo*	Mice (B6C3F1)	8–10	SC	Cysts	T45	30	35	Number of *Toxoplasma* cysts ± SD						
										RIA	Control		6	982 ± 194		
											DES		6	2244 ± 66^*^	**↑**	**<0.05**
											17β-estradiol		6	1934 ± 198^*^	**↑**	**<0.05**
											5α-Dihydrotest**i** osterone		6	792 ± 164	**↓**	
											Progesterone		6	1012 ± 172	**↑**	
											Zeralanol		6	1463 ± 190	**↑**	
											a-Dienestrol		6	2405 ± 227^*^	**↑**	**<0.05**
											Corticosterone		6	1954 ± 314^*^	**↑**	**<0.05**
										Effect of Tamoxifen, number of cysts ± SD						
										RIA	17β-estradiol (E2)	Control	6	1115 ± 112		
												Tamoxifen	6	975 ± 124	**↓**	
												17β-estradiol	6	2220 ± 182^*^	**↑**	**<0.05**
												Tamoxifen + E2	6	1027 ± 167	**↓**	
4	Fredriksson et al., 1990	*In vivo*	Ewes (Scottish blackface)	NS	Oral	Oocysts	RH	2000	90.5	Progesterone levels (nM/L)						
										RIA	Progesterone (P4)	Control	3	10–20		
												Infected	13	10	↓	NS
												Vaccinated	15	10	↓	NS
5	Aiumalamai et al., 1990	*In vivo*	Ewes (Swedish Peltsheep)	52–104	NS	Oocysts	NS	NS	90.5	Progesterone levels (nM/L)						
										RIA	Progesterone (P4)		7	Day 5: 6–8		
														**Days 10 a 15: 19-**	↑	**<0.05**
6	Hulínská et al., 1990	*In vivo*	Mice (H VUFB)	4–5	IP	Cysts	P78	10	Number of tachyzoites and stozoites							
									5–14	HIS y MIC	Cortisone	Group 1	20	10–14 days	**↑**	–
									12–47			Group 2	20			
7	Engeland et al., 1996	*In vivo*	Goat (Norwegian)	NS	SC	Bradyzoites	NS	1250	54–73	Progesterone levels						
										ELISA y SF	Progesterone (P4)	Control	6			
												Infected	5			
8	Stahl and Kaneda, 1998a	*In vivo*	Mice (Nya: NYLAR)	NS	IP	Cysts	CS	8	3 and 4	T4 levels (Mean)						
										RIA	Thyroxine (T4)	Control	10	7.5		
												Infected	10	**3**	↓	**<0.01**
9	Stahl and Kaneda, 1998a	*In vivo*	Mice (Nya: NYLAR)	12	IP	Cysts	CS	8	4	Subnormal T4 response to a 1 |ig bolus or TRH (Mean)						
										RIA	Thyroxine (T4)	Control	8	11		
												Infected	8	**3**	↓	**<0.01**
10	Liesenfeld et al., 2001	*In vivo*	Mice (C57BL/6)	8–10	Oral	Cysts	ME 49	100	7	Number of parasitophorous vacuoles						
										NS	Testosterone	Control		657 ± 399		
												Testosteron		**426 ± 282**	**↓**	**=0.0141**
11	Kaňková et al., 2011	*In vivo*	Mice (BALB/c and C57 Black)	5–6	Oral	Cysts	T38	10	60	Differences in serum testosterone levels						
										RIA	Testosterone	M. *Toxo* infected	12	***Z* = −2.32**	↓	**=0.005**
												M. Controls	20			
												F. *Toxo* infected	12	***Z* = −2.76**	↓	**=0.020**
												F. Controls	20			
12	Abdoli et al., 2012	*In vivo*	Rats (Wistar)	NS	IP	Tachyzoites	RH	1 × 10^7^		Effect of *T. gondii* infection on Serum Testosterone (ST)						
									10	ELISA	Testosterone	Uninfected	5	0.6 ± 0.01		
									10			Infected	3	0.55 ± 0.02^*^	↓	**<0.05**
										Effect of *T.gondii* infection on IntratesticularTestosteron (ITT)						
									10			Uninfected	5	4.07 ± 0.02		
									10			Infected	3	3.89 ± 0.05^*^	↓	**<0.05**
13	Puvanesuaran et al., 2012	*In vivo*	Mice (Swiss)	3	Oral	Tachyzoites	RH	1 × 10^4^	4	Number of tachyzoites (Mean)						
										MIC	Prednisolone	Control	3	1.48 × 10^7^		
												235 mg/kg	3	2.75 × 10^7^	**↑**	**<0.05**
												470 mg/kg	3	2.92 × 10^7^	**↑**	**<0.05**
												705 mg/kg	3	3.21 × 10^7^	**↑**	**<0.05**
14	Lim et al., 2013	*In vivo*	Rats (Wistar)	7	IP	Tachyzoites	PRU	5 × 10^6^	42–56	% Increase of Testosterona levels						
										ELISA	Testosterone		54	60%	**↑**	=0.057
15	Mitra et al., 2013	*In vivo*	Rats	6.5	IP	Tachyzoites	PRU	10 × 10^6^	42–56	Circuling levels of corticosterone						
										ELISA	Corticosterone		126	**64%**	↓	**<0.05**

a *SC, Subcutaneously; IP, Intraperitoneally; NA, Not Applicable*.

b *Type of strain: BK, Beverley; PRU, Prugniaud; CS, Cornell; RH, ME49, T45, P78, T38*.

c *HIS, Histological; RIA, Radioimmunoassay; MIC, Microscopical; SF, Sabin and Feldman; ELISA, Enzyme-Linked ImmunoSorbent Assay*.

d *E2, 17β-estradiol; P4, Progesterone; T4, Thyroxine; DES, Diethylstilbestrol; ST, Serum Testosterone; ITT, Intra testicular testosterone; TRH, Thyrotropin-Releasing Hormone*.

e *M, Male; F, Female; Gdt, Gonadectomy; Hex, Hexoestrol*.

f **↑**, Increased infection; **↓**, Decrement infection; ↑, Increased hormone; ↓, Decrement hormone;

* and bold, Statistically Significant. NS, Not specified; SD, Standard deviation.

**Table 3:** **Effect of *Toxoplasma gondii* infection on hormones in cell cultures**.

**References**		**Type of Study**	**Type of cell culture^a^**	**Stage parasite**	**Strain^b^**	**Number of parasites**	**Days post-infection**	**Analysis technique^c^**	**Hormone^d^**	**Group**	***N***	**Results^e^**		***p***
1	Benedetto et al., 2001	*In vitro*	MGC (C57BL/6)	Tachyzoites	RH	1 × 10^4^	20 h	Intracellular replicaton of *T. gondii (Mean* ± *SD*)						
								ELISA	Prolactin	Control		7.4 ± 1.0		
									(PRL)	PRL + rTNF-a		6.1 ± 1.0	**↓**	<0.05
2	Gay-Andrieu et al., 2002	*In vitro*	RAW 264.7	Tachyzoites	RH	3.3 × 10^6^	3–20 h	*Toxoplasma gondii* replication						
								IF, FC	Progesterone			No significant differences		<0.05
								y MIC						
3	Gets and Monroy, 2005	*In vitro*	RAW 264.7	Tachyzoites	RH	5 × 10^5^	18–24	Percentage of infected macrophages						
								MIC	Adrenaline	Control				
										Adrenaline *a*		5.55^*^	**↑**	<0.05
										Adrenaline p		10^*^	**↑**	<0.05
4	Jones et al., 2008	*In vitro*	BmSCs	Tachyzoites	RH	2 ×10^6^	1	Effect on LPS-induces killing on *T. gondii*						
								NS	Progesterone	Control		No significant differences		<0.05
										Infected				
5	Dzitko et al., 2010	*In vitro*		Tachyzoites	BK	2 x 10^5^		Influence of rhPRL en la intensidad de multiplication de *T.gondii*						
			L929				6	MTT	Prolactine			No significant differences		
			Hs27							1000.0 (ng/mL)	18	8.90 ± 3.46^*^	**↓**	**<0.01**
			HeLa									(No Sig. Diff.)		
								Inhibition of the proliferatio*n* rate (%) of *T. gondii*						
			L929				0 (min)			2.0–100.0 (ng/m L)	12	No significant differences		
							30			20.0 (ng/mL)	12	19.87 ± 4.28^*^	**↑**	**<0.05**
										100.0 (ng/mL)	12	23.66 ± 10.99^*^	**↑**	**<0.05**
							60			20.0 (ng/mL)	12	19.66 ± 5.73^*^	**↑**	**<0.01**
										100.0 (ng/mL)	12	25.53 ± 3.19^*^	**↑**	**<0.01**
							180			20.0 (ng/mL)	12	26.76 ± 3.02^*^	**↑**	**<0.01**
			Hs27				0 (min)			100.0 (ng/mL)	12	27.00 ± 2.50^*^	**↑**	**<0.01**
										2.0–100.0 (ng/m L)	12	No significant differences		
							30			20.0 (ng/mL)	12	20.81 ± 4.21^*^	**↑**	**<0.01**
										100.0 (ng/mL)	12	21.93 ± 5.48^*^	**↑**	**<0.01**
							60			20.0 (ng/mL)	12	19.05 ± 2.63^*^	**↑**	**<0.01**
										100.0 (ng/mL)	12	23.01 ± 5.93^*^	**↑**	**< 0.01**
							180			20.0 (ng/mL)	12	21.14 ± 5.62^*^	**↑**	**<0.01**
										100.0 (ng/mL)	12	36.15 ± 11.53^*^	**↑**	**<0.01**
			HeLa				0 (min)			2.0–100.0 (ng/mL)	12	No significant differences		
							30			20.0 (ng/mL)	12	23.05 ± 4.97^*^	**↑**	**<0.01**
										100.0 (ng/mL)	12	31.74 ± 5.79^*^	**↑**	**<0.01**
							60			20.0 (ng/mL)	12	27.71 ± 7.42^*^	**↑**	**<0.01**
										100.0 (ng/mL)	12	31.71 ± 7.06^*^	**↑**	**<0.01**
							180			20.0 (ng/mL)	12	29.64 ± 6.23^*^	**↑**	**<0.01**
										100.0 (ng/mL)	12	32.12 ± 3.53^*^	**↑**	**<0.01**
6	Dzitko et al., 2012	*In vitro*	PBMC	Tachyzoites	BK	2.5 × 10 5	3	% of *T. gondii* proliferation						
								ELISA	rhPRL	0 (ng/mL)		76.35 ± 10.1		
										100.0 (ng/mL)		81.01 ± 11.6		
									sPRL	0 (ng/mL)		49.8 ± 4.6		
										100.0 (ng/mL)		59.6 ± 3.1^*^	**↓**	**<0.01**
7	Dzitko et al., 2013	*In vitro*	L929	Tachyzoites		1 × 10 7		% increse of prolactine Levels						
					RH		30 (min)	ELISA	shPRL			10.1		
							90 (min)				NS	52.4	↑	=0.056
					ME49		30 (min)					16		
							90 (min)				NS	46.2	↑	=0.056

a *MGC, Microglial cell cultures; RAW 264.7, Murine Macrophage cell line; BmSCs, Bone marrow Stem Cells; L929, Mouse fibroblasts cell line; Hs27, Human foreskin fibroblast; HeLa, Human epithelial cells; PBMC, Peripheral Blood Mononuclear Cells*.

b *Type of strain: Beverley (BK), RH, ME49*.

c *MIC, Microscopical; IF, Immunofluorescence; FC, Flow Cytometry; ELISA, Enzyme-Linked ImmunoSorbent Assay; MTT, 3-(4,5-dimethylthiazol-2-yl)-2,5-diphenyltetrazolium bromide*.

d *PRL, Prolactin; rhPRL, Recombinant Human Prolactin; sPLR, Serum Prolactin; shPRL, Sheep Prolactin*.

e **↑**, Increased infection; **↓**, Decrement infection; ↑, Increased hormone; ↓, Decrement hormone;

* and bold, Statistically Significant. NS, Not specified; SD, Standard deviation.

### Humans

Eight articles were performed with different hormones on humans, from 17 to 40 years old: Testosterone (*n* = 5) (Oktenli et al., 2004; Hodková et al., 2007; Flegr et al., 2008a,b; Shirbazou et al., 2011), 17β-estradiol and progesterone, dehydroepiandrosterone (DHEA), prolactin, and cortisol and testosterone (*n* = 1) (Dzitko et al., 2008; Al-warid and Al-qadhi, 2012; de la Torre et al., 2012). These studies used Radioimmunoassay (RIA) or Enzyme-linked ImmunoSorbent assay (ELISA) in 8 studies combined with other analytic methods (Table 1).

### Animals

Fifteen articles evaluated the hormone effect in *T. gondii* infection using different animal models: murine model (*n* = 12); in guinea-pigs (1) (Kittas and Henry, 1979), in mice (8) (Kittas and Henry, 1980; Pung and Luster, 1986; Hulínská et al., 1990; Stahl and Kaneda, 1998a,b; Liesenfeld et al., 2001; Kaňková et al., 2011; Puvanesuaran et al., 2012), and rats (3) (Abdoli et al., 2012; Lim et al., 2013; Mitra et al., 2013). Two from ewes (2) (Aiumalamai et al., 1990; Fredriksson et al., 1990) and one for goats (1) (Engeland et al., 1996) (Table 2).

Progesterone and testosterone were the most studied hormones (*n* = 4), estradiol (*n* = 3), corticosterone and thyroxine (*n* = 2) and cortisone, adrenaline, and prednisolone (*n* = 1). Eight *T. gondii* strains were also analyzed: two Type I (eight RH and four BK) and six Type II (two PRU, ME49 and SC and one T45, P78, T38) and two not specified (Table 2).

The most frequent parasite stage of development studied was the tachyzoite (*n* = 11), followed by cyst (*n* = 8), ooquiste (*n* = 2), and bradizoite (*n* = 1). The number of parasites used for each experiment depended on the stage of parasite development and the host. In the murine model, tachyzoites from 1 × 10^4^ to 1 × 10^7^ were used (Benedetto et al., 2001; Abdoli et al., 2012; Dzitko et al., 2013). The number of cysts used in different rodent species was from 8 to 100 (Stahl and Kaneda, 1998b; Liesenfeld et al., 2001). In an experiment with goats, 1250 bradyzoytes were used (Engeland et al., 1996) and in another study with sheep infected with ooquistes, the number of ooquistes was not indicated (Aiumalamai et al., 1990) (Table 2).

The post-infection time in each experiment was different, according to each species and parasite stage of development. In guinea pigs, 42 days (Kittas and Henry, 1979); mice, 4 to 60 days (Kittas and Henry, 1980; Pung and Luster, 1986; Hulínská et al., 1990; Stahl and Kaneda, 1998a,b; Liesenfeld et al., 2001; Kaňková et al., 2011; Puvanesuaran et al., 2012); in rats, 10 to 56 days (Abdoli et al., 2012; Lim et al., 2013; Mitra et al., 2013), in a goat, 54 to 73 days (Engeland et al., 1996) and in ewes 90.5 days (Aiumalamai et al., 1990; Fredriksson et al., 1990) (Table 2).

Concerning the route of infection, 15 studies were carried out, four subcutaneous (Kittas and Henry, 1979, 1980; Pung and Luster, 1986; Engeland et al., 1996) and six more by peritoneal administration (Hulínská et al., 1990; Stahl and Kaneda, 1998a,b; Abdoli et al., 2012; Lim et al., 2013; Mitra et al., 2013). In four studies, oral administration was used for infection (Fredriksson et al., 1990; Liesenfeld et al., 2001; Kaňková et al., 2011; Puvanesuaran et al., 2012) and one was not specified (Aiumalamai et al., 1990) (Table 2).

### Cell cultures

Seven studies were designed in cell lines; two in RAW 264.7 mouse cell lines (Gay-Andrieu et al., 2002; Gets and Monroy, 2005), one, in bone marrow stem cells (Jones et al., 2008) one in microglial cell cultures (Benedetto et al., 2001) and three with prolactin in Murine L929, Human Hs27, HeLa, and Peritoneal Blood Mononuclear cells (PBMC) (Dzitko et al., 2010, 2012, 2013; Abdoli et al., 2012) (Table 3).

Concerning non-steroid hormones, prolactin and thyroxine hormone have been studied. In this study, other non-steroid hormones such as growth hormone, parathyroid, corticotrophin, insulin and glucagon, luteinizing and follicle hormone, thyroid stimulating, human chorionic gonadotropin, antidiuretic, oxytocin, melanocyte stimulating, somatostatin, thyrotropin-releasing hormone, gonadotropin-releasing hormone, noradrenaline, adrenaline, melatonin, and triiodothyronine were not associated to *Toxoplasma* infection.

The laboratory analysis methods used were: Radioimmunoassay (RIA) (Pung and Luster, 1986; Aiumalamai et al., 1990; Kaňková et al., 2011). Enzyme-Linked Immunosorbent Assay (ELISA) (Engeland et al., 1996; Abdoli et al., 2012; Dzitko et al., 2012, 2013; Lim et al., 2013). A Morphological Method, (MM), Indirect Immunofluorescence (IFI), Flow Cytometry Analysis (CF) (Gay-Andrieu et al., 2002), Microscopy (Hulínská et al., 1990; Gay-Andrieu et al., 2002), in three histological studies (Kittas and Henry, 1979, 1980; Hulínská et al., 1990) and in two methods. Sabin and Feldman (SF) (Engeland et al., 1996) Inverse Reaction of Polymerase Chain and ELISA (Lim et al., 2013).

## Discussion

Congenital toxoplasmosis is one of the most significant burdens of *T. gondii* infection in humans. Both the maternal–fetal transmission and hormonal levels during pregnancy are poorly understood and yet, may play an important role during the course of the disease. In pregnant women with acute toxoplasmosis, low levels of progesterone and low levels of estrogens can induce severe infection caused by *T. gondii* (Al-warid and Al-qadhi, 2012). The changes in endocrine phenomena occurring during pregnancy, as well as the size and maturity of the placenta and the embryonic/fetal immune response definitely affect the ability to be protected from invasion or to fight infection (Ortiz-Alegría et al., 2010).

In pregnant women with toxoplasmosis, low levels of progesterone and estrogen can induce severe infection. Nevertheless, the mechanism is unknown (Al-warid and Al-qadhi, 2012). Current studies show that there weren't any statistically significant differences in progesterone levels between infected and uninfected women with *T. gondii*, although higher progesterone levels were observed in uninfected women compared to low level in infected women. Moreover, estrogen levels in both chronic and uninfected women did not exhibit significant differences, although infected women had a higher level, compared to uninfected women.

The study of 17β-estradiol in *T. gondii* infection began in 1979, when hexoestrol was administered to mice and increased the number of *T. gondii* cysts in muscle (Kittas and Henry, 1979). At the same time, the susceptibility to *T. gondii* infection increased in mice when pharmacological estrogen concentrations were used (Pung and Luster, 1986). Nevertheless, 35 years have passed since these experiments were performed and no further studies regarding 17β-estradiol mechanism in *T. gondii* infection have been reported.

Progesterone levels are reduced during pregnancy in sheep after infection by *T. gondii* (Aiumalamai et al., 1990; Fredriksson et al., 1990). This hormonal change could be contributing to the susceptibility to *T. gondii* infection in sheep.

In RAW 264.7 cells infected with tachyzoites of *T. gondii*, progesterone did not regulate the replication of parasites (Gay-Andrieu et al., 2002). However, bone marrow stem cells activated with Lippolysaccharide (LPS) and treated with progesterone, while infected with *T. gondii* tachyzoites, cells exhibited a significant reduction in parasite death compared to activated controls (Jones et al., 2008). These results suggest that progesterone can modulate the survival of parasites *in vitro*.

The results of this study showed that steroid hormones are the most studied toxoplasmosis interaction. However, the information has a great heterogeneity and is not comparable, due to their different experimental designs. For example, the progesterone has been studied in mice (Pung and Luster, 1986), sheep (Aiumalamai et al., 1990), goats (Engeland et al., 1996), and bone marrow stem cells cultures (Jones et al., 2008). Furthermore, in these experiments, different strains and parasite stage of development were used. Moreover, no study has shown how steroid hormones regulate *T. gondii* infection.

The first observation of *T. gondii* infection and its association with testosterone in humans shows that acute infection by this parasite produced temporary hypogondatrophic gonadal insufficiency (Oktenli et al., 2004). On the other hand, there are several human studies analyzing different genders, using portrait pictures of 89 male students, of which 18 were *Toxoplasma* infected, and 109 female students. When statistically corrected for age, men with latent toxoplasmosis were perceived as more dominant (*p* = 0.009) and masculine (*p* = 0.052). These results suggest that the higher level of testosterone could be responsible for at least some of the toxoplasmosis-associated shifts in human and animal behavior (Hodková et al., 2007). In 2008, Flegr showed that the relationship between age, gender and 2D:4D ratio in hands sharply increased with *Toxoplasma* infection. Infected males had higher testosterone levels, while infected females had lower levels, than *Toxoplasma*-free males and females, respectively. *Toxoplasma*-infected males had a lower left hand 2D:4D ratio than *Toxoplasma*-free males. These results suggest that the relationship between 2D:4D ratio is particularly strong for the left hand and 2D:4D dimorphism will probably be higher in countries with a high prevalence of toxoplasmosis (Flegr et al., 2008b). These results indicate that sexual hormones and gender are key factors determining susceptibility to *Toxoplasma* infection.

Significantly, lower levels of testosterone in male and female mice with latent toxoplasmosis (strain T38 of *T. gondii*) were compared to uninfected controls (Kaňková et al., 2011). On the other hand, Liesenfeld in 2001 described the effect of sexual steroids and gender in the susceptibility to infection by *T. gondii* in mice. Death occurred in female mice before males, and mortality in females was associated to an increase in the number of tachyzoites. Female mice testosterone treatment reduced the number of parasites and pathology.

5α-Dihydrotestosterone reduced the number of cysts in mice infected with *T. gondii* cysts strain T45. Mice treated with corticosterone increased twice the number of cysts of *T. gondii* (Pung and Luster, 1986; Hulínská et al., 1990). These results showed that corticosterone could exacerbate the infection process.

The prevalence of *T. gondii* infection was analyzed in women with hyper and hypoprolactinemia, with a significant increase in this last group (Dzitko et al., 2008). In other studies using peripheral blood mononuclear cells (PBMC) of patients with hyperprolactinemia revealed that exogenous recombinant human prolactin (rhPRL), as well as autologous shPRL from inactivated serum, significantly restricted intracellular growth of *Toxoplasma* in these cultures (Dzitko et al., 2012). PRL may be one of the potential humoral factors implicated in the limitation of *T. gondii* invasion. A physiological increase in PRL concentration during pregnancy may significantly reduce the risk of *T. gondii* proliferating in the expecting mother (Dzitko et al., 2012).

rhPRL reduced *T. gondii* replication in human cells (Hs27 y HeLa) and murine cells (L929), (Dzitko et al., 2010, 2013). Afterwards in another experimental study, the replication of parasites was reduced in L929 cells treated with prolactin. These results indicate that the inhibition of replication of *T. gondii* was caused by a limited capacity of the parasites to penetrate host cells, as demonstrated by the reduced number of infected cells. On the other hand, PRL stimulates T cell proliferation (Clevenger et al., 1992) and the release of various protective cytokines as TNF-α which control efficiently the course of *T. gondii* infection (Benedetto et al., 2001). The possible PRL action could be bidirectional, namely PRL may limit the proliferation of *Toxoplasma* via surface host cell receptors (Dzitko et al., 2013) leading to the release of protective type-1 cytokines, such as interleukin 12 (IL-12) and IFN-c (Matalka, 2003), and by inhibiting their penetration ability (Dzitko et al., 2010, 2013).

In the last 35 years, researchers worldwide have made a great effort to advance in the field of knowledge on how the hormones are involved in *T. gondii* infection, however, a major number of studies and the use of modern molecular methods are required to define the mechanistic role of hormones in the regulation of toxoplasmosis.

### Implications for research

A crucial factor is the difference in experimental models to study of *T. gondii* infections and hormones. As well, type's strains and the number limited studies to comparative analysis.

### Conflict of interest statement

The authors declare that the research was conducted in the absence of any commercial or financial relationships that could be construed as a potential conflict of interest.
